# Editorial: Ontogeny and Phylogeny of Brain Barrier Mechanisms

**DOI:** 10.3389/fnins.2016.00041

**Published:** 2016-02-16

**Authors:** Helen B. Stolp, Shane A. Liddelow, Norman R. Saunders

**Affiliations:** ^1^Division of Biomedical Engineering and Health Sciences, Department of Perinatal Imaging and Health, King's College LondonLondon, UK; ^2^Pharmacology and Therapeutics, Developmental Neurobiology and Neurotrauma, University of MelbourneParkville, VIC, Australia; ^3^Neurobiology, Stanford UniversityWest Stanford, CA, USA

**Keywords:** blood-brain barrier, choroid plexus, tight junctions, influx mechanisms, efflux mechanisms, development, *Drosophila*, zebra fish

The protective barriers of the central nervous system, positioned at interfaces between the brain, spinal cord and the periphery, are often considered as gatekeepers—restricting entry of unwanted molecules and ensuring an effective maintenance of the delicate microenvironment of the central nervous system. While there is truth in this depiction, it does not fully capture the highly dynamic nature of these brain barrier systems; the specific regulation of transporters, the signaling between endothelial cells and other components of the neurovascular unit; or the precise regulation of cerebrospinal fluid composition via the choroid plexus, that changes appropriately both during development and throughout aging.

In this Frontiers Topic on *Ontogeny and phylogeny of brain barrier mechanisms*, we celebrate the advances made in understanding the normal structure and function of these mechanisms in a diverse range of organisms. The study of development and non-mammalian species shows the similarities of many brain barrier mechanisms as well some diversity.

Substantial information is now available about the protein composition of tight junctions, an integral component of barrier structure. As well as recent investigations on interaction of protein components, we are gaining insights into the polarity changes in endothelial and epithelial cells required for correct establishment of tight junctions (Bauer et al.). The polarization of cells at brain barriers is also key for regulation of transport, such has been clearly indicated for influx (Saunders et al.) and efflux transporters (Strazielle and Ghersi-Egea). The important transport role of the barrier systems in relation to brain development has been highlighted for thyroid hormone in the review by Richardson et al. Genomic studies highlight molecular diversity of the barriers, which show substantial changes in gene regulation with age, species and at different barrier sites (Bill and Korzh; Bueno et al. DeSalvo et al.; Johansson; Limmer et al.; Ek et al.; Saunders et al.; Strazielle and Ghersi-Egea). The use of non-mammalian animal models has advanced the capacity to interrogate the molecular diversity of the barrier systems (DeSalvo et al.; Henson et al.; Hindle and Bainton; Limmer et al.). Zebrafish in particular, because of their transparency, provide an unparalleled opportunity to observe directly at high resolution the development of particular aspects of barrier function (Bill and Korzh; Henson et al.). This plethora of species represents fantastic opportunities for further investigation of how regulation of gene or protein production affects barrier function.

The new genomic studies included in this Topic also report enrichment of genes not just for cell adhesion and solute transport, but also metabolism and cell signaling (DeSalvo et al.; Limmer et al.) indicate a more integrated, deterministic role of the barrier systems in brain function. This integration is particularly evident in neurogenic niches where proliferation and differentiation of cells are in part regulated by contact or release of signaling molecules from cells that comprise these barriers (Stolp and Molnár). To further emphasize this point, Errede et al. provide an elegant study showing cross-talk between blood vessels and radial glia within the developing human cerebral cortex.

For research into this complex biological system to advance further, it is necessary for a number of important concepts to be addressed. First, there needs to be consistent and accurate definitions of brain barrier systems. Second, recognition is required that barrier systems in development are not just present and functional, but also integral for facilitating normal brain growth. And third, there is an urgent need for identification and community-wide implementation of well-described, reproducible and meaningful assessments of barrier function.

Significant progress has been made regarding the first two of these concepts. In this special Topic Whish et al. and Brøchner et al. explore two under-studied barriers in the developing brain. These authors elegantly demonstrate multiple components of the barrier system at the inner and outer CSF-brain interfaces, and how these differ from blood-brain and blood-CSF barriers. Brøchner et al. extend on the barrier definitions outlined by Saunders et al. (2008). In particular they identify 3 distinct morphological components (arachnoid barrier cell layer, pial microvessels, and glial end feet/pial surface layer) that contribute to the outer CSF-brain barrier interface that has previously been treated as a single entity. Bueno et al. review experiments on very early stages of brain development shortly after neural tube closure in the chick embryo. This is a stage that has otherwise received very little attention. They describe a subset of endothelial cells in the ventral mesencephalon and anterior ventral prosencephalon with transient transport properties that they suggest parallel the functions of the choroid plexuses before these structures differentiate.

The misconception that brain barrier systems are immature in the developing brain is still frequently touted in the literature, despite evidence to the contrary dating back to experiments conducted in the early twentieth century (see Saunders et al.). In this special Topic, further evidence is presented on age- and region-specific regulation of influx and efflux transporters (Saunders et al.; Strazielle and Ghersi-Egea)—as described above, the developing brain actually has more individual barrier systems than in the adult (Bueno et al.; Brøchner et al.; Whish et al.). The choroid plexus in particular is a complex set of barrier mechanisms that are present from an extremely early stage of brain development (Liddelow). The evidence presented on progenitor cells specifically sitting in CSF and vascular niches points to a tightly controlled environment with specific signaling mechanisms appropriate for different stages of the development of the brain is particularly striking. This contradicts the long held immature brain barrier hypothesis (Bueno et al.; Johansson; Stolp and Molnár). There is also evidence for an additional neurogenic niche in the choroid plexus stroma (Prasongchean et al.), which these authors suggest contributes specifically to prenatal innervation of the choroid plexus and regulation of CSF secretion.

While there are huge advances in our understanding of structural (e.g., tight junction) proteins and transporter systems at brain barriers during development and aging in a wide variety of species, we are still limited in our understanding of how these elements of the barrier systems are altered following injury or during disease. The field is currently most interested in barrier dysfunction in the adult and aging brain, aspects that are outside the scope of this Topic. However, barrier dysfunction during development has implications not only for the fetus or newborn acutely at the time of the disorder but also chronically with the possibility of long-term consequences. Here Moretti et al. provide an overview of disorders that involve brain barrier mechanisms including their long-term sequelae. Kratzer et al. deal with the specific developmental problem of neonatal stroke; while Palmela et al. provide some *in vitro* data on the problem of neonatal kernicterus. The role of the blood-brain barrier in neonatal bilirubin encephalopathy is much more complex than the longstanding simple view that it occurs because of barrier absence or immaturity (see Saunders et al.).

Both in the developmental and adult brain barrier fields we are missing a consensus on the appropriate methodologies that should be used to understand barrier function. For example, quantification of tight junction proteins is a common assessment in injury models to determine whether structural elements of barriers are functioning effectively. However, there is no clear indication of (a) what effect reduction in tight junction proteins produces, or (b) how changes in expression of tight junction protein genes or cellular content of these proteins relate to structural integrity of junctions. To make claims about loss of barrier integrity requires ultrastructural studies with suitable markers that are visible at high resolution, something that is rarely done. If brain barrier studies are to integrate with a proper understanding of neurological injury we need substantial improvement in our assessments of barrier function. This focus on structural proteins of brain barriers also means that active transport systems, important for understanding of energetics of the barriers, are rarely studied. This is despite evidence of high metabolic function of cerebral endothelial cells compared to those in the periphery (Oldendorf et al., 1977). While tracer studies still represent a functional measure, irrespective of mechanism, there are substantial problems with how these have been administered and interpreted (see Saunders et al.).

Leukocyte infiltration into the brain during injury is often used as an indication of barrier dysfunction, but is a finding that is not interpreted so easily. Leukocytes cross into the brain under control conditions as part of the normal immune surveillance of the brain (Ransohoff and Engelhardt, 2012), but it is unclear whether the massive influx of leukocytes into the brain (e.g., in Multiple Sclerosis active lesions) is a dysfunction of the barrier, or a normal pathological response resulting from abnormal signaling in the brain. There is evidence from perinatal brain injury of leukocyte populations entering the brain and contributing to injury at a time when the adaptive immune system had previously been considered immature and unresponsive (Wang and Mallard, 2016). It is unclear whether some of these cells are also contributing to resolution of the lesion, or whether they only produce negative effects on the brain. Whatever the reason for peripheral immune cell infiltration, there is increasing evidence of beneficial effects of controlled neuroinflammation in pathological conditions (Mardiguian et al., 2013; Evans et al., 2014; Amantea and Bagetta, 2016).

Clear guidelines for conducting genomic or proteomic studies of barrier systems are now provided (Huntley et al.; Torbett et al.). What remains is for these studies to extend from an analysis of normal barrier interfaces, to studies of heterogeneity of barrier systems and of cerebral endothelial cells (Macdonald et al., 2010) as well as responses to injury and other pathological conditions. Similarly, there is need for a battery of standardized tests for barrier function, including tracers and other measures of barrier integrity as well as transporter presence and function. For a full understanding of normal and abnormal barrier function these should be performed together, in whole systems studies at macro- and microscopic levels. The wide range of molecular and physiological tools available, as well as the multiple *in vitro* and *in vivo* models, means that these aims are well within reach.

Two problems that have been a major preoccupation in the blood-brain barrier field over recent decades have been how to screen drugs with potential therapeutic value for treating neurological disorders, and how to devise methods for effective delivery of such drugs to the brain. In spite of a considerable expenditure of time and resources outcomes have been disappointing, perhaps in part because of an undue focus on *in vitro* methods. Although not a specific focus of this Topic, several of the papers point to contributions that are, or could be made by approaches described here. Thus, Hindle and Bainton describe promising live imaging approaches in *Drosophila*, supported by the report of DeSalvo et al. that the transcriptomes of fly and mouse show many similarities particularly with respect to the ABC efflux transporters that prevent the entry of so many drugs into the central nervous system. The advances in understanding of many different aspects of barrier function described in the papers in this Topic hold promise for developing better drug delivery systems both *in vivo* and in improved *in vitro* systems.

The twenty-five reviews and original research articles in this Topic, provide a comprehensive update on the state of these research fields, their interrelations and implications for the blood-brain barrier field as a whole. We should like to thank all the authors and additional editors for their splendid and patient contributions to the Topic. We hope that the Topic articles will provide both a benchmark and reference point for future studies.

## Author contributions

All authors listed, have made substantial, direct and intellectual contribution to the work, and approved it for publication.

### Conflict of interest statement

The authors declare that the research was conducted in the absence of any commercial or financial relationships that could be construed as a potential conflict of interest.
